# Supplementary material for: Prefrontal cortex neurons encode ambient light intensity differentially across regions and layers
Source: Nat Commun. 2024 Jun 29;15:5501. doi: 10.1038/s41467-024-49794-w (PMC11217280; doi:10.1038/s41467-024-49794-w)
Supplement: Supplementary file 1 — Supplementary Information [file 41467_2024_49794_MOESM1_ESM.pdf]

Prefrontal cortex neurons encode ambient light intensity differentially across regions and layers

Elyashiv Zangen[1], Shira Hadar[1], Christopher Lawrence[1], Mustafa Obeid[1], Hala Rasras[1], Ella Hanzin[1], Ori Aslan[1], Eyal Zur[1], Nadav Schulcz[1], Daniel Cohen-Hatab[1], Yona Samama[1], Sarah Nir[1], Yi Li[1], Irina Dobrotvorskia[1], Shai Sabbah[1*]

[1] Department of Medical Neurobiology, Faculty of Medicine, The Hebrew University of Jerusalem, Jerusalem, 9112102, Israel

[*]Corresponding author

Contents:

Supplementary Figures 1-9

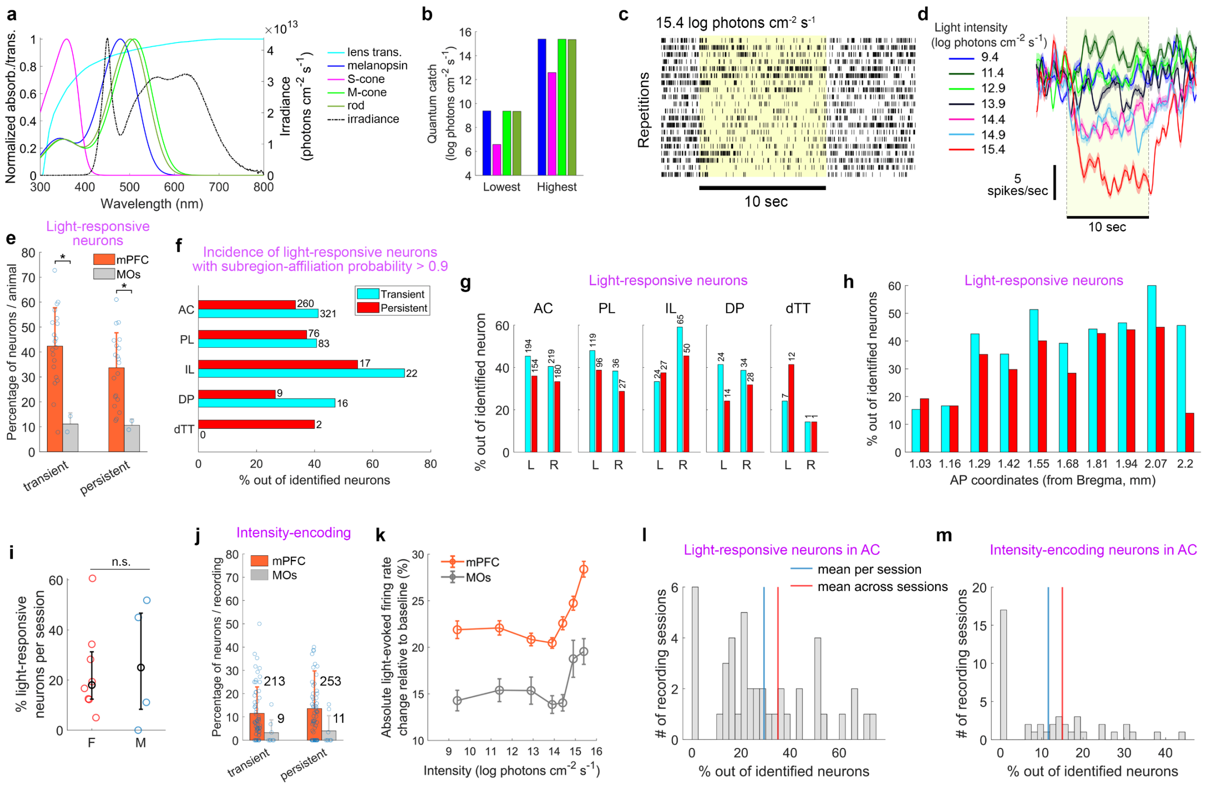

Supplementary Figure 1. Light stimulus and identification of transient and persistent light-responsive neurons of the mPFC and MOs

a, The stimulus irradiance spectrum (dashed line, right y-axis), spectral transmittance of the mouse lens, and spectral absorbances of the mouse rod, cone, and melanopsin pigments (solid lines, left y-axis), were used to estimate the quantum catches of the different pigments. **b,** Quantum catches of melanopsin, S-cone, M-cone, and rod are presented for the lowest and highest stimulus intensities. **c,d,** Light-evoked firing of an example neuron. Raster of light-evoked action potentials in response to a 15.4 log photons cm^{-2} s^{-1} stimulus intensity, over 20 repetitions (**c**), and the mean (±SEM) firing rate (FR) for the seven tested intensities (**d**). Light exposure suppressed this neuron's FR during the 10-sec stimulus. At high intensities, firing suppression persisted after stimulus termination. **e.** Percentage (mean ± SD, across animals) of mPFC neurons demonstrating transient and persistent light-responsiveness was significantly higher than in the MOs (permutation t-test, one-sided: p = 0.009 and 0.0029 for transient and persistent neurons; mPFC: 20 mice, MOs: 2 mice). **f,** Incidence of transient and persistent light-responsive neurons was significantly higher in the IL vs. the remaining subregions when accounting only for neurons with a probability larger than 0.9 to being affiliated with the respective subregion [transient: $\chi^2(1, 1107) = 5.008$, one-sided, p = 0.025; persistent: $\chi^2(1, 1107) = 6.999$, one-sided, p = 0.008]. **g,** Incidence of light-responsive neurons across hemispheres varied slightly, but not in a statistical-significant manner, between subregions. **h,** Incidence of light-responsive neurons varied along the mPFC's anterior-posterior axis. **i,** Incidence (median, 25th and 75th percentile) across recording sessions of persistent light-responsive neurons did not vary between the sexes (permutation t-test, two-sided: p = 0.795; 8 and 6 sessions in females and males, respectively), as did the incidence across animals (permutation t-test, two-sided: p = 0.30). The sex-documented data set included 301 and 133 neurons, acquired during 8 and 5 recording sessions, in 3 females and 3 males, respectively. Of these neurons, 70 and 53 were light-responsive, in females and males, respectively. **j,** Percentage (mean ± SD, across recording sessions) of transient and persistent intensity-encoding neurons in the mPFC was significantly larger than in the MOs (permutation t-test, one-sided: transient, p = 0.012, n = 60; persistent, p = 0.016, n = 8). **k,** Absolute light-evoked FR change from baseline (mean ± SEM across

all identified neurons) in mPFC was significantly higher than in the MOs, across all seven tested intensities (permutation t-test, one-sided, p adjusted for multiple comparisons; p = 0.0035, 0.0008, 0.0042, 1E-6, 1E-6, 0.0075, 1E-6, from the lowest to highest intensity). **l,m,** Variation in the incidence of light-responsive neurons (**l**) and intensity-encoding neurons (**m**) out of identified neurons per session. The vertical, coloured lines represent the mean incidence of these neurons out of the neurons identified per session (blue), and the mean incidence of these neurons out of the number of neurons identified across all sessions (red). Source data are provided as a Source Data file.

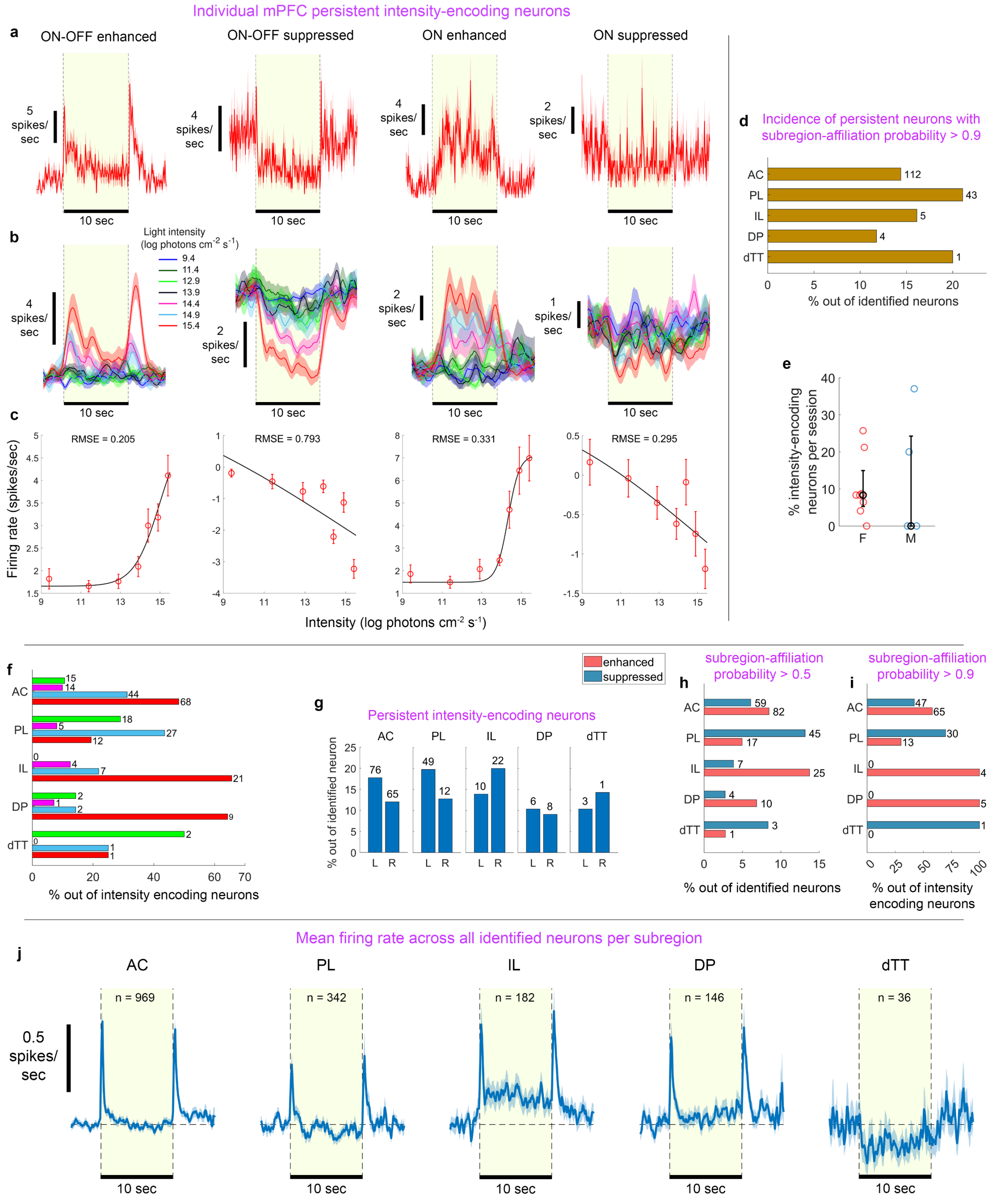

Supplementary Figure 2. Individual mPFC neurons encode light intensity

a-c, Representative neurons of the four identified functional types of persistent intensity-encoding neurons in the mPFC. **a,** Light-evoked FR (mean ± SEM) in response to the highest intensity (15.4 log photons cm^{-2} s^{-1}). **b,** Smoothed (binned at 10 points and linearly interpolated) light-evoked FR (mean ± SEM) in response to the 7 tested light intensities. While smoothing resulted in displacement of data points along the abscissa (intensity) axis, it facilitates the appreciation of FR differences across light intensities. **c,** Intensity-response (mean ± SEM) along with a sigmoid fit, and the resulting RMSE. **d,** While accounting only for neurons with subregion-affiliation probabilities > 0.9, the incidence of persistent intensity-encoding neurons was highest in the PL and IL [$\chi^2(1, 1107) = 7.00$, one-sided, p =

0.011]. **e,** Incidence (median, 25th and 75th percentile) across recording sessions of mPFC persistent intensity-encoding neurons did not differ between the sexes (permutation t-test, two-sided: $p = 0.893$), as did the incidence across animals (permutation t-test, two-sided: $p = 0.299$, 3 mice of each sex). Out of the 301 and 133 neurons identified in females and males, 29 and 26 were intensity-encoding, respectively. **f,** Distribution of the four intensity-encoding types across the mPFC subregions. **g,** Incidence of intensity-encoding neurons across hemispheres varied slightly, but not statistically-significantly, between subregions. **h,** Also when their incidences were calculated out of all identified neurons, the IL and DP were dominated by the two enhancement-response types, and the PL and dTT by the two suppression-response types [IL vs. PL: $\chi^2(1, 93) = 22.90$, one-sided, $p = 0.0005$; DP vs. dTT: $\chi^2(1, 18) = 2.82$, one-sided, $p = 0.09$]. **i,** While accounting only for neurons with subregion-affiliation probabilities > 0.9, the IL and DP were dominated by the two enhancement-response types, while the PL and dTT by the two suppression-response types [IL vs. PL: $\chi^2(1, 60) = 12.59$, one-sided, $p = 0.0035$; DP vs. dTT: $\chi^2(1, 5) = 5.00$, $p = 0.025$]. **j,** Mean (± SEM) light-evoked FR of all the neurons identified, including light-responsive and non-responsive neurons, across the mPFC subregions. Source data are provided as a Source Data file.

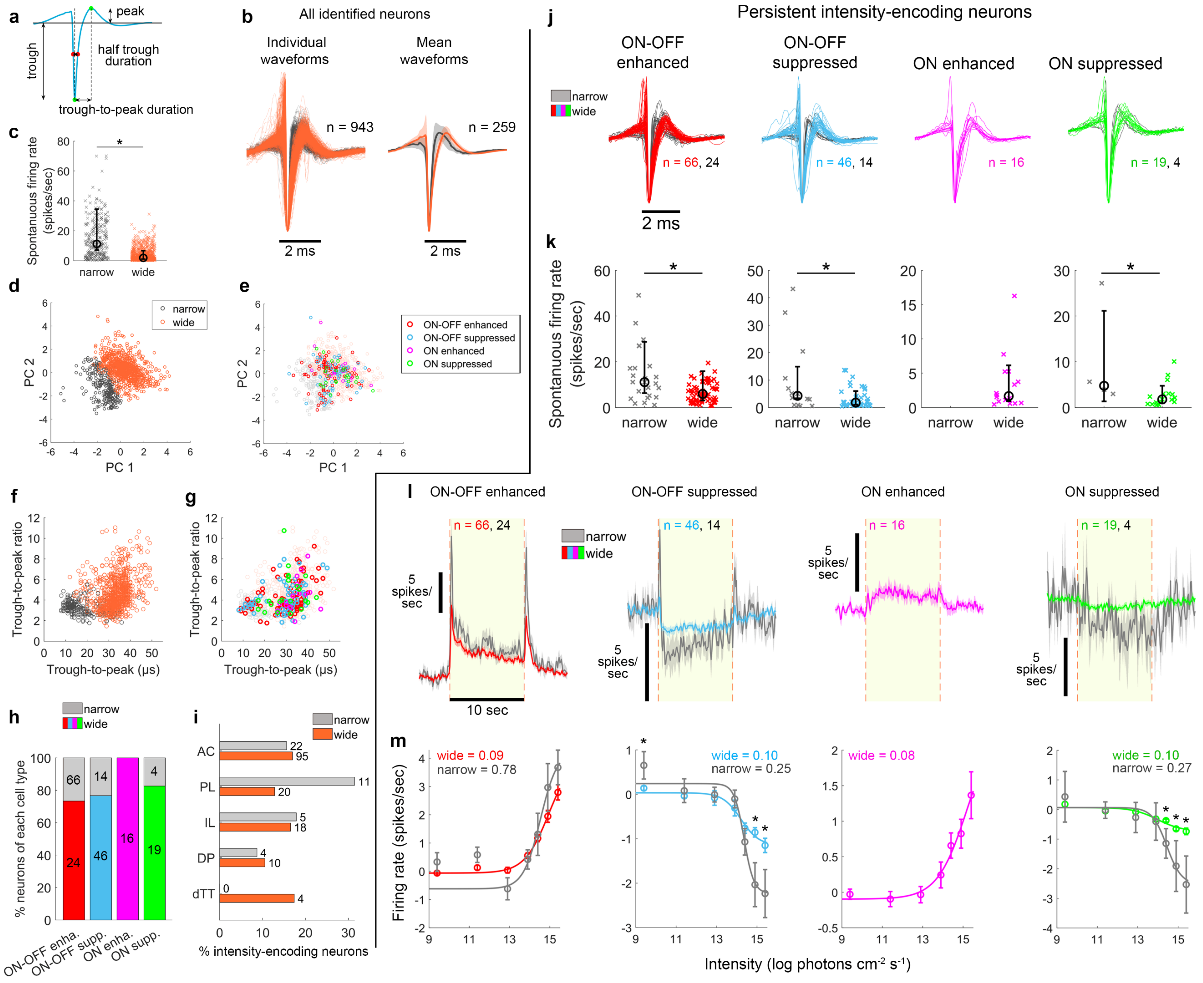

Supplementary Figure 3. Persistent intensity-encoding mPFC neurons correspond to presumptive pyramidal neurons and interneurons

a, Definition of four key electrophysiological properties used for analysis: spontaneous FR, trough-to-peak duration, trough-to-peak ratio, and trough half-duration. **b-c,** Principal component analysis followed by hierarchical clustering on the four electrophysiological properties distinguished between two mPFC neuronal populations, corresponding to presumptive pyramidal excitatory neurons (wide waveform, low FR) and inhibitory interneurons (narrow waveform, high FR); this distinction is consistent with previous reports[88-90]. Spontaneous FR of the narrow- and wide-waveform neuronal populations differed significantly [permutation t-test, two-sided, $p = 0.0003$; median (25th, 75th percentile): 'narrow': 11.3 (4.2, 23.3) spikes/sec, 'wide': 1.9 (0.8, 4.8) spikes/sec]. **d,f,** The distinction between excitatory and inhibitory neurons is evident when plotting the first two principal components (**d**), and even more so when plotting the waveforms' trough-to-peak duration against their trough-to-peak ratio (**f**). **e,g,** Overlap between the four response types and the two neuronal types (excitatory and inhibitory). Red, blue, magenta, green circles: individual neurons of the four response types. Orange, grey circles: individual excitatory and inhibitory neurons. 'ON-OFF enhanced', n = 90; 'ON-OFF suppressed', n = 60; 'ON enhanced', n = 16; 'ON suppressed', n = 23. **h,** Distribution of the four response types across the excitatory and inhibitory neuronal types. E_{on} neurons corresponded only with presumptive excitatory pyramidal neurons. However, each of the remaining response types represented a mixture of presumptive excitatory pyramidal neurons and inhibitory interneurons (73%-83% matched an excitatory pyramidal neuron profile), in agreement with previous reports of the relative incidence of the two cortical neuronal types [89,100]. **i,** Percentage of excitatory and inhibitory neurons that encode light intensity in each mPFC subregion. Presumable interneurons (31%) were more than twice as prevalent as presumable pyramidal neurons (13%) in the PL, while having similar proportions in the AC, IL, and

DP. **j,k,** The two distinct neuronal populations were apparent when inspecting the action potential waveforms of the four response types, divided into the two neuronal types (**j**), and when inspecting the spontaneous FR of intensity-encoding mPFC neurons belonging to individual functional types, for each of the two neuronal types (permutation t-test, two-sided; 'ON-OFF enhanced', $p = 0.0004$; 'ON-OFF suppressed', $p = 0.0035$; 'ON suppressed', $p = 0.0327$; asterisks mark $p < 0.05$) (**k**). **l,m,** Light-evoked FR as a function of time (**l**) and light intensity (**m**), for the four response types, divided into the two neuronal types. Light-evoked FR differed significantly between the two neuronal types, only at high intensities and only for the 'ON-OFF suppressed' and 'ON suppressed' types (permutation t-test, two-sided, p adjusted for multiple comparisons, 'ON-OFF suppressed': $p = 0.05$ and 0.004, for highest and 2nd highest intensities; 'ON suppressed': $p = 0.017$, 0.042, and 0.041, for highest and 2nd and 3rd highest intensities; asterisks mark intensities for which < 0.05). Source data are provided as a Source Data file.

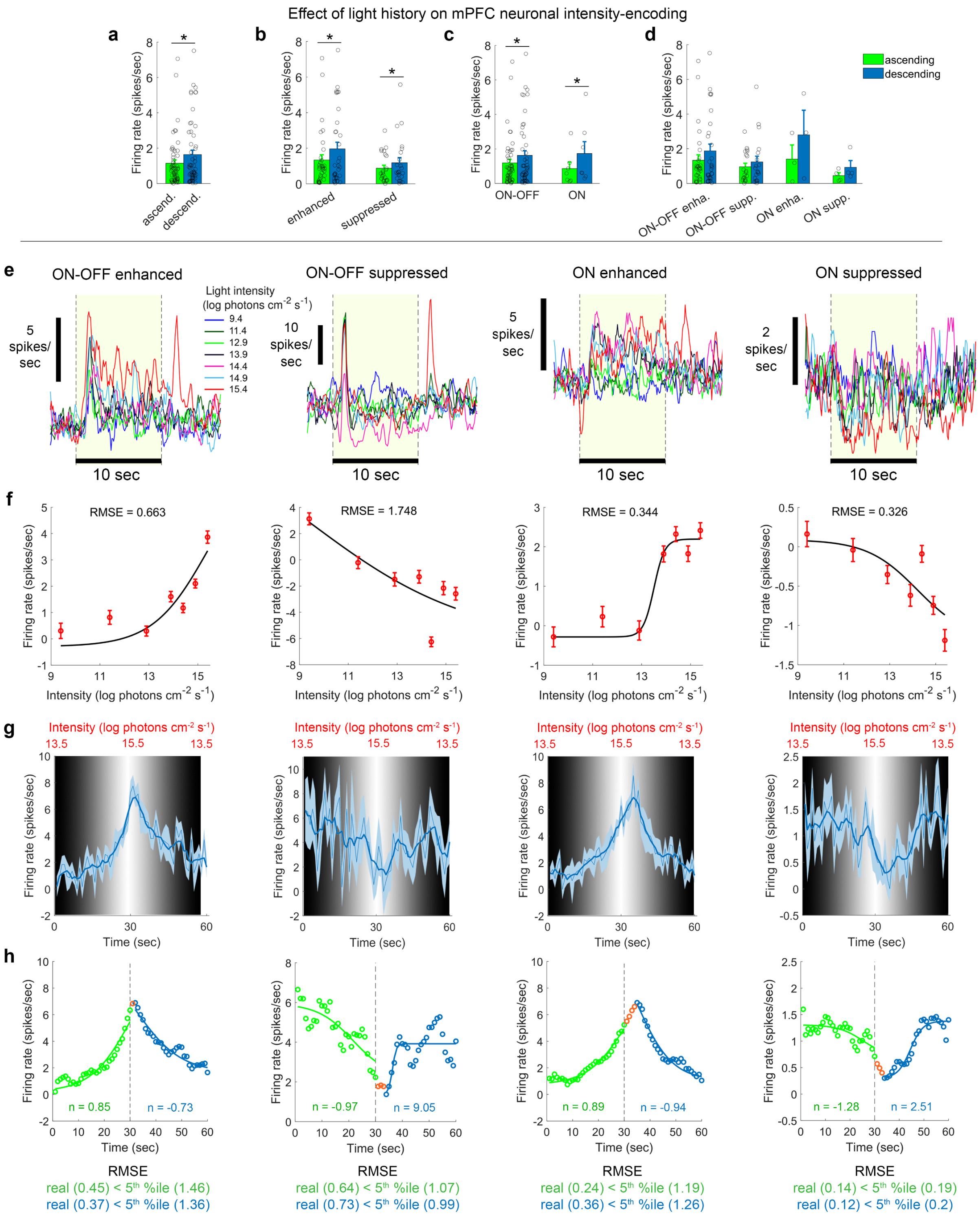

Supplementary Figure 4. Light history affects mPFC firing, and firing rate of individual neurons follows intensity gradients

a-d, FR over the 7 sec after the transition time (when stimulus intensity exceeds 15 log photons cm^{-2} s^{-1}) was significantly higher than FR over the 7 sec prior the transition time (permutation t-test, one-sided, p=0.0001 for neurons of all types pooled, n=55) (**a**). The effect of prior light exposure on FR around the transition time was significant also when probing the 'enhanced' (p=0.022, n=32) and 'suppressed' (p=0.002, n=23) types separately (**b**), and when probing the 'ON-OFF' (p=0.002, n=48) and 'ON'

(p=0.008, n=7) types separately (**c**), but not when probing each type separately, probably due to a limited sample size (**d**). **e,** Light-evoked FR (mean, across 20 repetitions) in response to the 7 tested light intensities, for neurons representative of the four functional types. **f,** Steady-state FR (mean ± SEM, across 20 repetitions) as a function of light intensity for the four intensity-encoding types. **g,** FR modulations (mean ± SEM, across 20 repetitions) of the four functional types in response to the bi-phasic stimulus (continuously ascending and then descending intensity). The secondary (top) abscissa and the grayscale gradient under the curves represent the light intensity gradient. A smoothed version (thick line; moving average, 5 sec) of the raw, noisy, FR trace (thin line) is presented. **h,** Sigmoid fit to FR encountered throughout each phase, for representatives of each of the four types. The transition time (30 sec) is marked by a vertical dashed black line; slopes (*n*) of the sigmoid fit to the ascending and descending phases are indicated; $RMSE_{real}$ and the 5th percentile of $RMSE_{null}$ indicated below plots. Orange data points: timepoints in which the ascending and descending phases in FR lagged behind the transition between the intensity ascending and descending phases. Source data are provided as a Source Data file.

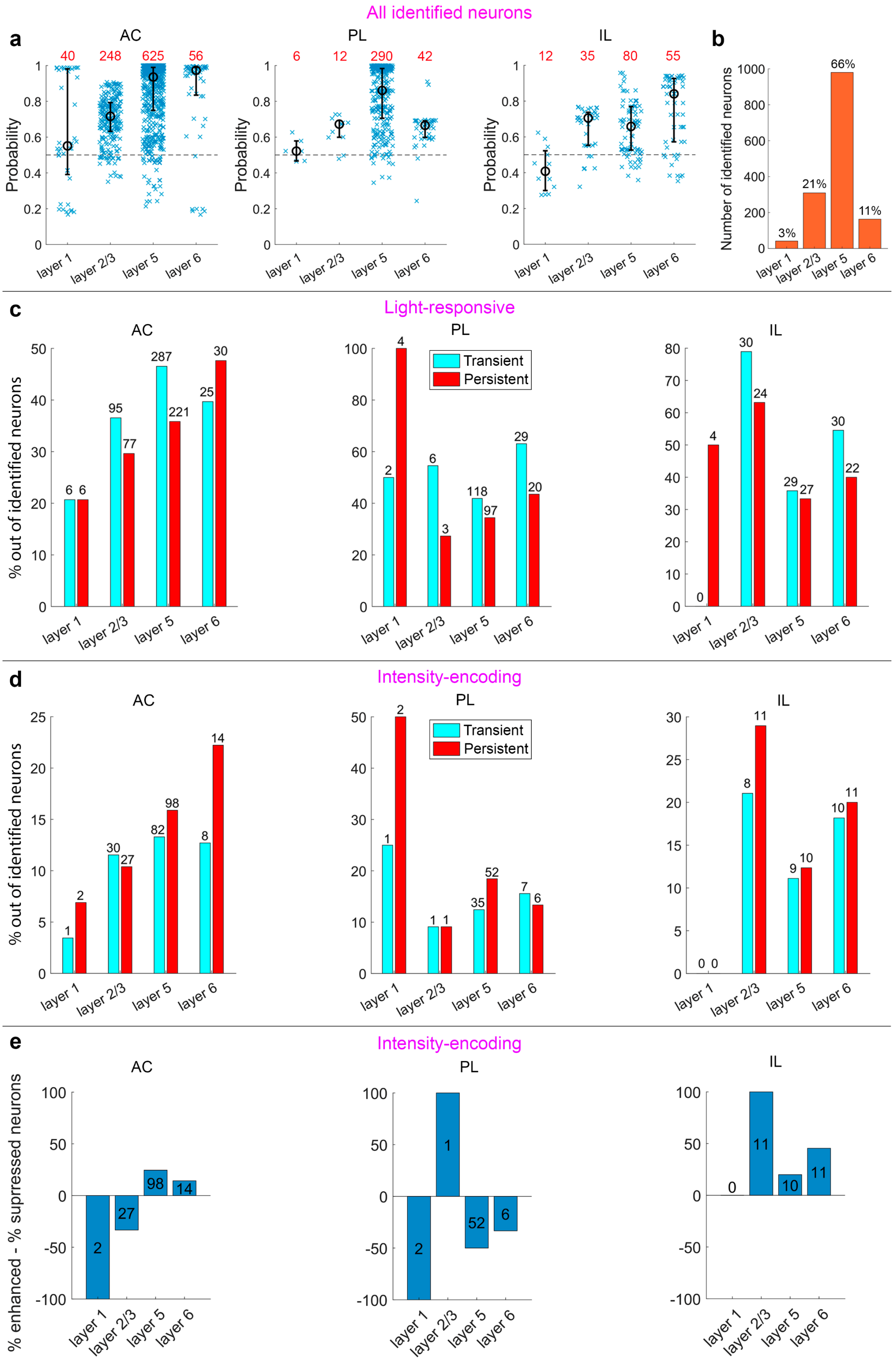

Supplementary Figure 5. Incidence of light-responsive and intensity-encoding neurons across mPFC layers

a, Probability of identified neurons to be located within the assigned cortical layer, in the AC, PL, and IL (numbers of neurons assigned to each cortical layer are presented). Probability of neurons to be assigned to layer 1 (L1), in all three mPFC subregions, was low, and therefore no interpretation of L1

data was attempted. **b,** Total number of neurons identified in the AC, PL, and IL, across layers. **c,** Percentages of transient and persistent light-responsive neurons across the different layers in the AC, PL, and IL. The incidence of transient and persistent light-responsive neurons differed significantly between L2/3 and L5/6 in the AC [transient, $\chi^2(1, 940) = 6.68$, $p = 0.009$; persistence, $\chi^2(1, 940) = 4.41$, $p = 0.03$] and IL [transient, $\chi^2(1, 174) = 15.03$, $p = 0.001$; persistent, $\chi^2(1, 174) = 8.97$, $p = 0.003$], but not in the PL [transient, $\chi^2(1, 338) = 0.39$, $p = 0.529$; persistent, $\chi^2(1, 338) = 0.31$, $p = 0.575$]. **d,** Percentages of transient and persistent intensity-encoding neurons across the different layers in the AC, PL, and IL. The incidence of transient intensity-encoding neurons did not differ significantly between L2/3 and L5/6 either in the AC [$\chi^2(1, 940) = 0.486$, $p = 0.485$], IL [$\chi^2(1, 174) = 1.14$, $p = 0.286$], or PL [$\chi^2(1, 338) = 0.13$, $p = 0.713$]. In contrast, the incidence of persistent intensity-encoding neurons differed significantly between L2/3 and L5/6 in the AC [$\chi^2(1, 940) = 5.53$, $p = 0.018$], but not in the IL [$\chi^2(1, 174) = 3.61$, $p = 0.057$] and PL [$\chi^2(1, 338) = 0.55$, $p = 0.457$]. **e,** Incidence of 'enhanced' relative to that of 'suppressed' intensity-encoding neurons across layers, for the AC, PL, and IL. The relative incidence of 'enhanced' and 'suppressed' neurons differed significantly between L2/3 and L5/6 in the AC [$\chi^2(1, 139) = 7.06$, $p = 0.008$] and IL [$\chi^2(1, 32) = 4.69$, $p = 0.03$], but not in the PL [$\chi^2(1, 59) = 2.73$, $p = 0.09$]. All χ^2 tests are one-sided. Source data are provided as a Source Data file.

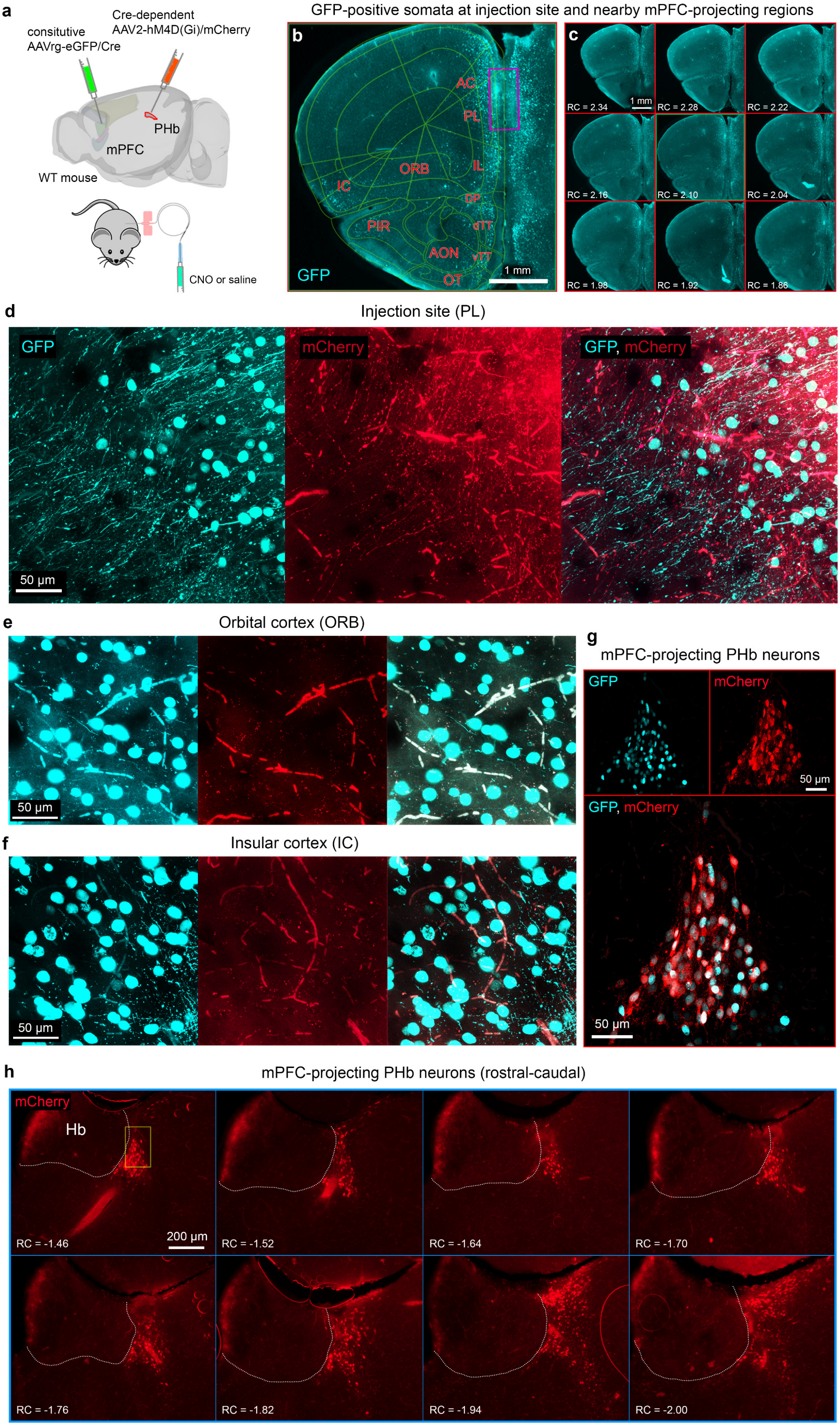
a
consitutive
AAVrg-eGFP/Cre
Cre-dependent
AAV2-hM4D(Gi)/mCherry
PHb
mPFC
WT mouse
CNO or saline
GFP-positive somata at injection site and nearby mPFC-projecting regions
b
AC
PL
IL
ORB
IC
DP
PIR
dTT
AON
vTT
OT
GFP
1 mm
c
1 mm
RC = 2.34
RC = 2.28
RC = 2.22
RC = 2.16
RC = 2.10
RC = 2.04
RC = 1.98
RC = 1.92
RC = 1.86
d
Injection site (PL)
GFP
mCherry
GFP, mCherry
50 µm
e
Orbital cortex (ORB)
50 µm
f
Insular cortex (IC)
50 µm
g
mPFC-projecting PHb neurons
GFP
mCherry
50 µm
GFP, mCherry
50 µm
h
mPFC-projecting PHb neurons (rostral-caudal)
mCherry
Hb
200 µm
RC = -1.46
RC = -1.52
RC = -1.64
RC = -1.70
RC = -1.76
RC = -1.82
RC = -1.94
RC = -2.00

Supplementary Figure 6. Mapping mPFC-projecting PHb neurons

a, Bilateral injection of a retrograde Cre/GFP-expressing AAV in the mPFC (in this example, in the PL), and a Cre-dependent AAV-DREADD/mCherry in the PHb, led to expression of DREADD/mCherry specifically in PFC-projecting PHb neurons. **b,** GFP-positive somata are present at the retrograde AAV injection site (magenta rectangular) and in other brain regions that project to these regions, including the infralimbic cortex (IL), dorsal peduncular area (DP), dorsal and ventral taenia tecta (TTd,v), orbital cortex (ORB), insular cortex (IC), and anterior olfactory nucleus (AON). GFP-positive somata were also present in the piriform cortex (PIR), a source of mPFC input identified here for the first time. **c,** A series of 9 mPFC slices along the rostral-caudal (RC) axis, with coordinates relative to bregma. The central slice (RC = 2.1mm) is the one shown in panel (**b**). **d,** mCherry-positive axons are present at the injection site. In addition to labelling axons, mCherry also labelled blood vessels that appear thicker than axons (for unknown reasons, Cre-dependent mCherry-labelled axons are poorly labelled with GFP). **e-f,** mCherry-positive axons are absent in the orbital cortex (**e**) or insular cortex (**f**). **g,** Co-localized GFP and mCherry fluorescence mark mPFC-projecting PHb neurons. **h,** Example of mCherry-positive, mPFC-projecting PHb neurons, along the rostral-caudal axis, with coordinates relative to bregma. The yellow frame, top-right panel, is the one shown in panel (**g**).

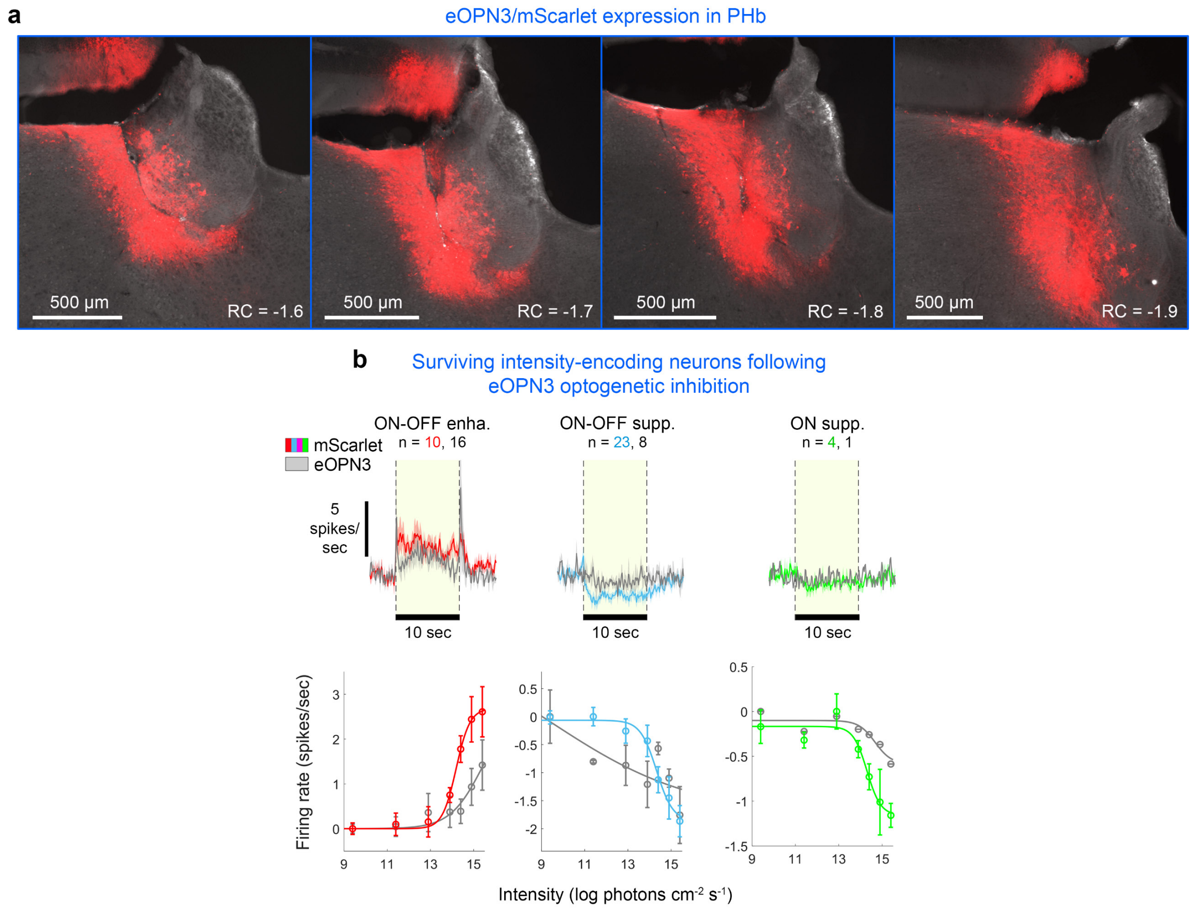

Supplementary Figure 7. Optogenetic inhibition of PHb transmission to mPFC

a, Example of eOPN3/mScarlet-positive PHb neurons, along the rostral-caudal axis, with coordinates relative to bregma. The left-most image is similar to **Fig. 4f**. **b,** FR (mean ± SEM) over time in mice expressing eOPN3/mScarlet (grey) vs. mScarlet (coloured) of the four persistent, intensity-encoding types, in response to the highest intensity (15.4 log photons cm^{-2} s^{-1}) (**top**), and steady-state FR (mean ± SEM) as a function of light intensity along the sigmoid fit (**bottom**).

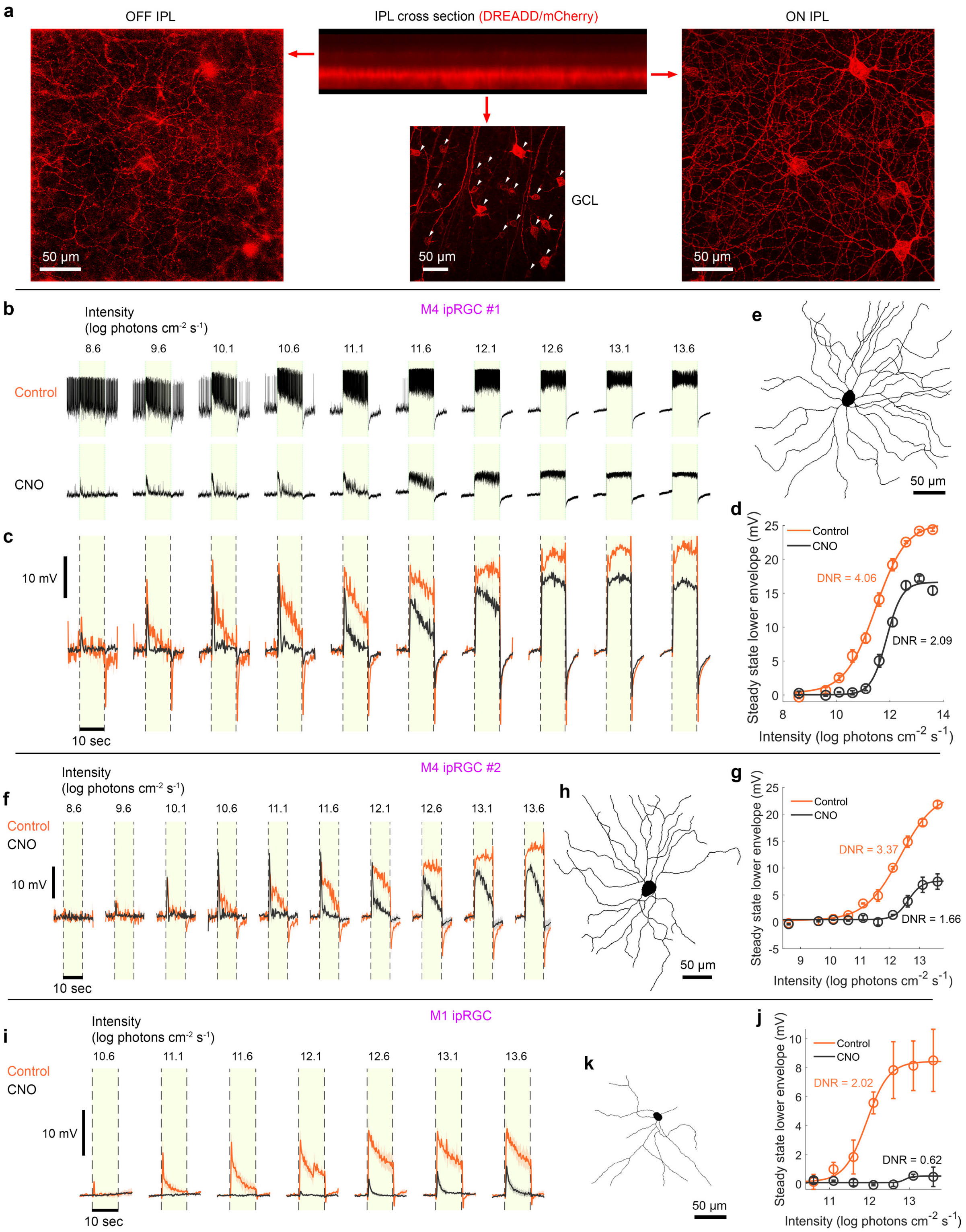

Supplementary Figure 8. Chemogenetic inhibition of ipRGCs

a, Intravitreal AAV injection led to inhibitory DREADD/mCherry expression in the OFF sublamina of the retina's inner plexiform layer (IPL), where M1, M3, and M6 ipRGCs stratify[15,101,102], as well as in the ON sublamina of the IPL, where M2, M3, M4, M5 and M6 ipRGCs stratify[18,36,103,104]. The relatively dense OFF IPL plexus suggests that labelled neurites include dendrites of M6 cells in addition to dendrites of M1 and/or M3 cells. The diverse soma sizes encountered in the ganglion cell layer (GCL), from ~10 µm (M1) to ~25 µm (M4), suggests the expression of DREADD/mCherry is multiple ipRGC types. It is unknown which ipRGC types contribute to mPFC photosensitivity, via the PHb or other

brain regions. However, previous retrograde transsynaptic tracing showed that ipRGCs feed the PHb-mPFC pathway [10], and retrograde tracing showed that the PHb is innervated by the M1 and M4 ipRGC types [10,38]. Thus, we focused the validation of chemogenetic inhibition on M1 and M4 cells. **b-e,** Whole-cell patch-clamp voltage recordings from an inhibitory DREADD-expressing M4 ipRGC in a flat-mount retina, before (control) and after adding CNO to the Ames solution. **b,** Light-evoked firing from an M4 ipRGC in response to 10 light intensities (8.6 - 13.6 log photons cm^{-2} s^{-1}), before and after CNO. **c,** Light-evoked membrane voltage under the 10 tested light intensities. The lower voltage envelop was calculated because this and other cells typically reached a depolarization block at the high stimulus intensities, precluding a meaningful estimation of FR. At high light intensities, membrane voltage retained its persistence after CNO. **d,** Steady-state intensity-response curve (mean ± SEM across 5 stimulus repetitions), along the fitted sigmoid, before and after CNO. CNO decreased the steady-state (over the 5 last sec of stimulus) membrane voltage and its dynamic range (DNR) across which the cell encoded intensity (DNR is given in log photons cm^{-2} s^{-1}; see *Methods* for DNR estimation). **e,** Morphological statistics of the M4 ipRGC were consistent with previous reports: soma diameter = 21.03 μm, dendritic field diameter = 323.51 μm, total dendritic length = 5284 μm, number of branch points = 32, number of primary dendrites = 6. **f-h,** Voltage recording from another M4 ipRGC that expresses an inhibitory DREADD. **f,** The response at high light intensities partially lost its persistence after CNO. **g,** CNO decreased membrane voltage across all tested intensities, and the DNR over which the cell encoded intensity narrowed. **h,** Morphological statistics of this M4 ipRGC were consistent with previous reports: soma diameter = 23.64 μm, dendritic field diameter = 323.36 μm, total dendritic length = 4829 μm, number of branch points = 32, number of primary dendrites = 6. **i-k,** Same as (**f-h**) but for an M1 ipRGC. **i,** Membrane voltage under 7 tested light intensities, before (control) and after adding CNO to the Ames solution. **j,** CNO decreased membrane voltage and abolished the capacity for intensity encoding. **k,** Morphological statistics of the M1 ipRGC were consistent with previous reports: soma diameter = 12.47 μm, number of branch points = 7, number of primary dendrites = 4 (dendritic field diameter and total dendritic length could not be calculated because the dendritic field was not imaged in its entirety). Source data are provided as a Source Data file.

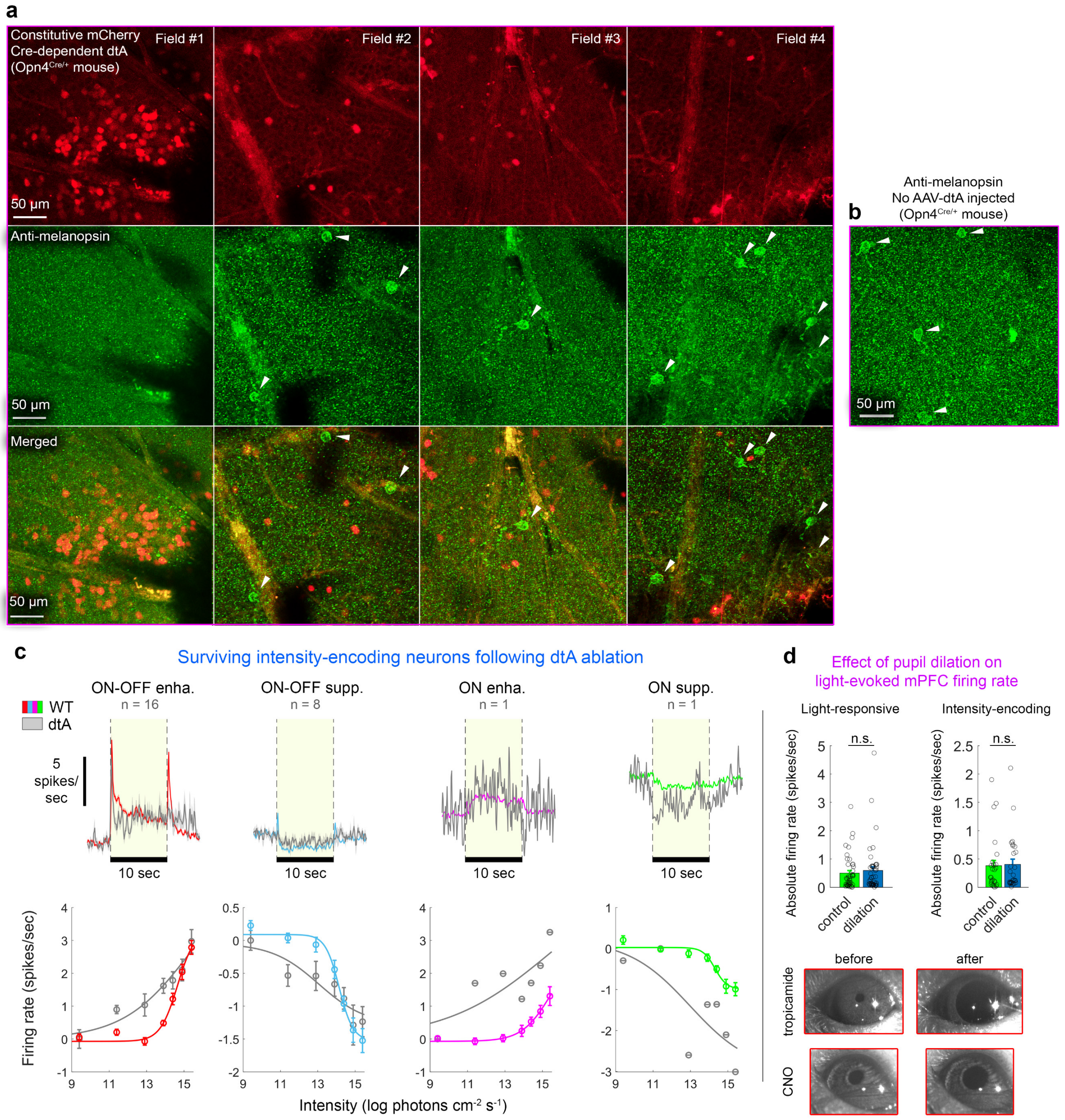

Supplementary Figure 9. Terminal ablation of ipRGCs and effect of pupil dilation on firing rate of mPFC neurons

a, Retinal mCherry expression and melanopsin immunoreactivity. Retinal regions exhibiting high mCherry expression (and thus also high dtA expression) did not contain any melanopsin-positive ipRGCs (field #1). However retinal regions with low mCherry expression (and thus also low dtA expression) included a small number of melanopsin-positive ipRGCs, suggesting the ablation of a fraction of ipRGCs (fields #2-4). **b,** Melanopsin immunoreactivity in an Opn4$^{Cre/+}$ mouse that has not been injected with the AAV-dtA is equally prevalent as in retinal regions with low mCherry expression in AAV-dtA-injected mice. **c,** FR (mean ± SEM) over time in AAV-dtA-injected mice (grey) vs. control WT mice (coloured) of the four persistent, intensity-encoding types, in response to the highest intensity (15.4 log photons cm^{-2} s^{-1}) (**top**), and steady-state FR (mean ± SEM) as a function of light intensity along the sigmoid fit (**bottom**). **d.** Pharmacologically dilating the pupils using tropicamide did not affect absolute FR of light-responsive (**left**) or intensity-encoding (**right**) mPFC neurons (paired permutation

t-test, two-sided; light-responsive: p = 0.186, n = 44 neurons; intensity-encoding: p = 0.397, n = 27 neurons). Source data are provided as a Source Data file.

Supplementary references

100 Xing, B., Morrissey, M. D. & Takehara-Nishiuchi, K. Distributed representations of temporal stimulus associations across regular-firing and fast-spiking neurons in rat medial prefrontal cortex. *J. Neurophysiol.* **123**, 439-450 (2020). https://doi.org/10.1152/jn.00565.2019

101 Quattrochi, L. E. *et al.* The M6 cell: A small-field bistratified photosensitive retinal ganglion cell. *J. Comp. Neurol.* **527**, 297-311 (2019). https://doi.org/10.1002/cne.24556

102 Schmidt, T. M. & Kofuji, P. Structure and function of bistratified intrinsically photosensitive retinal ganglion cells in the mouse. *J. Comp. Neurol.* **519**, 1492-1504 (2011). https://doi.org/10.1002/cne.22579

103 Stabio, M. E. *et al.* The M5 cell: A color-opponent intrinsically photosensitive retinal ganglion cell. *Neuron* **97**, 250-163 (2018). https://doi.org/10.1016/j.neuron.2017.12.030

104 Sabbah, S. *et al.* Intrinsically photosensitive retinal ganglion cells evade temporal filtering to encode environmental light intensity. *bioRxiv* (2022). https://doi.org/10.1101/2022.04.09.487733
