# Supplementary material for: Prefrontal cortex neurons encode ambient light intensity differentially across regions and layers
Source: Nat Commun. 2024 Jun 29;15:5501. doi: 10.1038/s41467-024-49794-w (PMC11217280; doi:10.1038/s41467-024-49794-w)
Supplement: Supplementary file 3 — Reporting Summary [file 41467_2024_49794_MOESM3_ESM.pdf]

nature portfolio

Corresponding author(s): Shai Sabbah

Last updated by author(s): May 24, 2024

Reporting Summary

Nature Portfolio wishes to improve the reproducibility of the work that we publish. This form provides structure for consistency and transparency in reporting. For further information on Nature Portfolio policies, see our Editorial Policies and the Editorial Policy Checklist.

Statistics

For all statistical analyses, confirm that the following items are present in the figure legend, table legend, main text, or Methods section.

n/a	Confirmed	
☐	☒	The exact sample size (*n*) for each experimental group/condition, given as a discrete number and unit of measurement
☐	☒	A statement on whether measurements were taken from distinct samples or whether the same sample was measured repeatedly
☐	☒	The statistical test(s) used AND whether they are one- or two-sided<br>*Only common tests should be described solely by name; describe more complex techniques in the Methods section.*
☒	☐	A description of all covariates tested
☐	☒	A description of any assumptions or corrections, such as tests of normality and adjustment for multiple comparisons
☐	☒	A full description of the statistical parameters including central tendency (e.g. means) or other basic estimates (e.g. regression coefficient) AND variation (e.g. standard deviation) or associated estimates of uncertainty (e.g. confidence intervals)
☐	☒	For null hypothesis testing, the test statistic (e.g. *F*, *t*, *r*) with confidence intervals, effect sizes, degrees of freedom and *P* value noted<br>*Give P values as exact values whenever suitable.*
☒	☐	For Bayesian analysis, information on the choice of priors and Markov chain Monte Carlo settings
☒	☐	For hierarchical and complex designs, identification of the appropriate level for tests and full reporting of outcomes
☐	☒	Estimates of effect sizes (e.g. Cohen's *d*, Pearson's *r*), indicating how they were calculated

Our web collection on statistics for biologists contains articles on many of the points above.

Software and code

Policy information about availability of computer code

Data collection	In vivo extracellular recordings were performed using OmniPlex Server version 1.17.1.0 software (Plexon).<br>Visual stimulus was created by custom code within Matlab (2020b and later MathWorks Inc)<br>In vitro patch clamp recordings were preformed using a Multiclamp 700B amplifier, Digidata 1550 digitizer, and pClamp 10.5 data acquisition software (Molecular Devices)

Data analysis

In vivo electrophysiological recordings were analyzed using:
Offline Sorter version 4.4.0.0, Plexon.
Kilosort 3 (https://github.com/MouseLand/Kilosort)
Phy2 (https://github.com/cortex-lab/phy)
SHARP-Track (https://github.com/cortex-lab/allenCCF)
Costume code in MATALB (2020b and later MathWorks Inc), and Python (v 3.7.6 or higher).

The code generated during this study is available at: https://github.com/elyashivzangen/light-and-the-mPFC-code (doi.org/10.5281/zenodo.11265775).

For manuscripts utilizing custom algorithms or software that are central to the research but not yet described in published literature, software must be made available to editors and reviewers. We strongly encourage code deposition in a community repository (e.g. GitHub). See the Nature Portfolio guidelines for submitting code & software for further information.

Data

Policy information about availability of data

All manuscripts must include a data availability statement. This statement should provide the following information, where applicable:

- Accession codes, unique identifiers, or web links for publicly available datasets
- A description of any restrictions on data availability
- For clinical datasets or third party data, please ensure that the statement adheres to our policy

We provided source data for Main and Supplementary Figures. The electrophysiological data after spike sorting associated with this study are available in figshare: "Prefrontal cortex neurons encode ambient light intensity differentially across regions and layers", with the identifier data DOI:"10.6084/m9.figshare.23659974" and is available at the following URL https://figshare.com/s/cf9dd54f122789dcd4a5
The raw electrophysiological files (.pl2) are available from corresponding author upon request.

Research involving human participants, their data, or biological material

Policy information about studies with human participants or human data. See also policy information about sex, gender (identity/presentation), and sexual orientation and race, ethnicity and racism.

Reporting on sex and gender

Use the terms sex (biological attribute) and gender (shaped by social and cultural circumstances) carefully in order to avoid confusing both terms. Indicate if findings apply to only one sex or gender; describe whether sex and gender were considered in study design; whether sex and/or gender was determined based on self-reporting or assigned and methods used.
Provide in the source data disaggregated sex and gender data, where this information has been collected, and if consent has been obtained for sharing of individual-level data; provide overall numbers in this Reporting Summary. Please state if this information has not been collected.
Report sex- and gender-based analyses where performed, justify reasons for lack of sex- and gender-based analysis.

Reporting on race, ethnicity, or other socially relevant groupings

Please specify the socially constructed or socially relevant categorization variable(s) used in your manuscript and explain why they were used. Please note that such variables should not be used as proxies for other socially constructed/relevant variables (for example, race or ethnicity should not be used as a proxy for socioeconomic status).
Provide clear definitions of the relevant terms used, how they were provided (by the participants/respondents, the researchers, or third parties), and the method(s) used to classify people into the different categories (e.g. self-report, census or administrative data, social media data, etc.)
Please provide details about how you controlled for confounding variables in your analyses.

Population characteristics

Describe the covariate-relevant population characteristics of the human research participants (e.g. age, genotypic information, past and current diagnosis and treatment categories). If you filled out the behavioural & social sciences study design questions and have nothing to add here, write "See above."

Recruitment

Describe how participants were recruited. Outline any potential self-selection bias or other biases that may be present and how these are likely to impact results.

Ethics oversight

Identify the organization(s) that approved the study protocol.

Note that full information on the approval of the study protocol must also be provided in the manuscript.

Field-specific reporting

Please select the one below that is the best fit for your research. If you are not sure, read the appropriate sections before making your selection.

- [x] Life sciences
- [ ] Behavioural & social sciences
- [ ] Ecological, evolutionary & environmental sciences

For a reference copy of the document with all sections, see nature.com/documents/nr-reporting-summary-flat.pdf

Life sciences study design

All studies must disclose on these points even when the disclosure is negative.

Sample size	To establish the minimum detectable effect size in $\chi 2$ tests, we conducted a prior sensitivity analysis using the G*Power 3 program (version 3.1.9.4) with power (1-beta) of 0.8 and an alpha error rate of 0.05.<br><br>The main data set of mPFC recordings included 1682 single neurons captured throughout 60 recording sessions, in 20 mice. This sample size ensured a large enough number of captured neurons in each of the five subregions of the mPFC, encompassing the majority of each subregion volume.<br><br>The data set of recordings in response to the combined 7-intensity and bi-phasic stimuli included 431 mPFC neurons. This sample size was sufficient for testing whether mPFC neurons continuously modulate their firing rate in response to light intensity gradients.<br><br>The data set of recordings in the PHb included 410 neurons captured throughout 16 recording sessions in 9 mice. This sample size enabled identifying the different functional neuronal types harboring the PHb.<br><br>The data set of recordings from the mPFC before and after chemogenetically inhibiting mPFC-projecting PHb neurons included 39 neurons captured throughout 2 recording sessions, in 2 mice. This sample size enabled testing the contribution of transmission from the PHb on mPFC photosensitivity.<br><br>The data set of recordings from the mPFC before and after chemogenetically inhibiting ipRGCs included 445 neurons captured throughout 18 recording sessions, in 7 mice. This sample size was sufficient for testing the effect of ipRGC inhibition on light responsiveness and intensity-encoding in the mPFC.<br><br>The data set of recordings from the mPFC following terminal ablation of ipRGCs included 473 neurons. The control mCherry group included 158 neurons. These sample sizes were sufficient for testing the effect of ipRGC ablation on light responsiveness and intensity-encoding in the mPFC.<br><br>The data set of recordings from the mPFC before and after optogenetically inhibiting mPFC-projecting PHb neurons included 221 neurons. The control mScarlet group included 145 neurons.
Data exclusions	In the main mPFC data set, neurons that were mapped outside of the mPFC (based on SHARP-track analysis) were excluded.<br>In the classification of mPFC neurons into excitatory vs. inhibitory types, to prevent any non-neuronal activity or poor neuronal readings from affecting the calculation of trough-to-peak time and the clustering routine, waveforms exhibiting (1) a peak smaller than a trough (exonal spikes), (2) low amplitude, or (3) no peak or a trough, were excluded from analysis.<br><br>For PHb recordings, neurons captured during recording sessions in which the electrode did not overlap with the volume of the PHb, were excluded from analysis.<br><br>For mPFC recording before and after chemogenetic inhibition of the mPFC-projecting PHb neurons, mice in which any of the four viral vector injections was inaccurate, were excluded from analysis. Additionally, any recording session during which a movement of the electrode array was identified (based on drift calculations in Kilosort) were excluded from analysis.<br><br>For mPFC recording before and after chemogenetic inhibition of ipRGCs, any recording session during which a movement of the electrode array was identified (based on drift calculations in Kilosort) were excluded from analysis.
Replication	The number of mice used in each experiment, and the number of recording sessions performed in each experiment, are specified in the Sample Size section above.
Randomization	All mice used in this study were randomly allocated to the different experiments.
Blinding	Investigators were not blinded to group allocation during data collection. Investigators were not blinded to group allocation during data analysis which was largely procedural, as it was performed computationally and without offering the investigator any meaningful interpretation of the results until the final analysis steps.

Reporting for specific materials, systems and methods

We require information from authors about some types of materials, experimental systems and methods used in many studies. Here, indicate whether each material, system or method listed is relevant to your study. If you are not sure if a list item applies to your research, read the appropriate section before selecting a response.

Materials & experimental systems

n/a	Involved in the study
	☒ Antibodies
☒	Eukaryotic cell lines
☒	Palaeontology and archaeology
	☒ Animals and other organisms
☒	Clinical data
☒	Dual use research of concern
☒	Plants

Methods

n/a	Involved in the study
☒	ChIP-seq
☒	Flow cytometry
☒	MRI-based neuroimaging

Antibodies

Antibodies used

Rabbit anti-melanopsin, ab19306, Abcam
Goat Anti-Rabbit IgG H&L (Alexa Fluor® 488) preadsorbed, ab150081, Abcam

Validation

Primary antibody - Tested applications: WB, IHC-P, ICC/IF, species reactivity: Mouse, Rat, Human

Animals and other research organisms

Policy information about studies involving animals; ARRIVE guidelines recommended for reporting animal research, and Sex and Gender in Research

Laboratory animals

Male and female adult (2-4 months old; 23-30 g) WT mice (C57BL/6J, Jackson Laboratory) and Opn4Cre/+ mice expressing Cre recombinase in ipRGCs (a generous gift from David Berson). Mice were housed at a temperature of 22°C in groups of 3-4, with food and water ad libitum and available nesting/enrichment material.

Wild animals

The study did not involve wild animals.

Reporting on sex

Sex information has been collected for a small data set (3 females and 3 males) but not for the main data set.

Field-collected samples

The study did not involve samples collected from the field.

Ethics oversight

All experimental procedures were approved by the Authority for Biological and Biomedical Models at the Hebrew University.

Note that full information on the approval of the study protocol must also be provided in the manuscript.
